# Serbian Healthcare Students’ Perceptions of and Readiness to Care for People with Intellectual Disabilities: A Cross-Sectional Study

**DOI:** 10.3390/healthcare13111315

**Published:** 2025-06-01

**Authors:** Dragana Milutinović, Dragana Simin, Katarzyna Ćwirynkało, Monika Parchomiuk, Zdzisław Kazanowski, Agnieszka Żyta, Špela Golubović

**Affiliations:** 1 Department of Nursing, Faculty of Medicine, University of Novi Sad, 21000 Novi Sad, Serbia; 2 Institute of Pedagogical Sciences, University of Warmia and Mazury in Olsztyn, 10-719 Olsztyn, Poland; k.cwirynkalo@uwm.edu.pl (K.Ć.); agnieszka.zyta@uwm.edu.pl (A.Ż.); 3 Institute of Pedagogy, Faculty of Pedagogy and Psychology, Maria-Curie Sklodowska University, 20-704 Lublin, Poland; monika.parchomiuk@mail.umcs.pl (M.P.); zdzislaw.kazanowski@mail.umcs.pl (Z.K.); 4 Department of Special Education and Rehabilitation, Faculty of Medicine, University of Novi Sad, 21000 Novi Sad, Serbia; spela.golubovic@mf.uns.ac.rs

**Keywords:** intellectual disability, opinions, social distancing, professional competence, medical students, nursing students, questionnaires

## Abstract

**Background/Objectives:** The perspective from which future healthcare professionals view intellectual disabilities affects how people with intellectual disabilities (PWIDs) are perceived and informs care policies and practices. This study aimed to assess healthcare science students’ perceptions of the rights of PWIDs, the students’ social distances toward PWIDs in healthcare, and the students’ competence in providing care, exploring differences by study programs and demographics and examining correlations between them. **Methods:** The convenience sample comprised 221 medical and 120 nursing students. A general questionnaire for obtaining sociodemographic data, the scale of beliefs about the rights of PWIDs in healthcare (BS), the scale of social distance toward PWIDs (SD), and the “self-assessment of competency (CS) to provide care for PWIDs” scale were used as students’ report measures. **Results:** The students’ scores on the BS, SD, and CS scales revealed that they generally recognized the rights of PWIDs in healthcare but expressed a moderate level of social distance and limited self-perceived competence in providing care. Medical students demonstrated slightly more progressive beliefs regarding the rights of PWIDs than nursing students (r = 0.12), while nursing students reported higher self-assessed competence levels (r = 0.19). A small gender-related difference was observed in social distance, with female students showing more favorable attitudes. Significant positive correlations were found between beliefs about the rights of PWIDs and social distance (*p* = 0.435; *p* < 0.01) and between social distance and self-assessed competence (*p* = 0.234, *p* < 0.01), suggesting that students who felt more competent tended to report less social distance. **Conclusions:** This study provides new data for understanding healthcare science students’ perceptions and readiness to care for PWIDs in the healthcare sector in Serbia. Namely, our students had moderately positive beliefs and a moderate social distance toward PWIDs and reported low competence in providing care.

## 1. Introduction

Despite the United Nations’ Convention on the Rights of Persons with Disabilities, emphasizing equality and civil and human rights for individuals with intellectual disabilities, including the right to adequate healthcare, self-advocacy, therapy and rehabilitation, community living, and education, numerous factors still hinder the realization of these rights in practice [1]. Namely, people with intellectual disabilities (PWIDs) face significant challenges within the healthcare system, such as a lack of understanding of their needs, negative attitudes and discrimination from healthcare professionals, insufficient knowledge and information, and inadequate data collection and analysis on disability-related issues, like mortality and morbidity rates, all of which contribute to health inequities faced by this group [2,3,4].

Recent studies have identified numerous obstacles that impede PWIDs from obtaining quality healthcare services [5,6]. These include architectural and urban challenges, a lack of resources, and inadequately trained professionals. The absence of protocols or guidelines adapted to their specific needs further complicates their access to health services [5]. However, healthcare professionals’ prejudices and biases, resulting from a lack of knowledge, insufficient skills to conduct health assessments and address needs, and discomfort in caring for or communicating with PWIDs, significantly impact the quality of service provided and lead to low self-image and self-confidence and negative health outcomes for PWIDs [6].

Therefore, there is a pressing need for a comprehensive educational framework that offers continuous learning opportunities at different proficiency levels to ensure healthcare professionals can effectively meet their diverse needs. Although conflicting findings exist regarding whether attitudes toward PWIDs are influenced by experience and knowledge about PWIDs [7,8,9], it has been shown that health professionals who frequently provide care for patients with disabilities, such as those in rehabilitation, have more positive attitudes than their colleagues who do not have such experience [10].

In developing countries, like Serbia, medicine and nursing school curricula often provide only basic information on the causes and treatment of various disabilities, presenting disabilities primarily from a pathological perspective and neglecting a holistic approach to health and the basic human rights of people with disabilities [10]. Consequently, there is a lack of information on effective communication with PWIDs, providing information and establishing trusting relationships, necessary accommodations, and the rights of and opportunities available to PWIDs [10,11]. A similar situation exists in most European countries, except for the United Kingdom and Ireland. Namely, these countries offer basic programs leading to the qualification of registered nurses for caring for people with intellectual disabilities/learning disabilities, preparing them to provide specialized person-centered care for PWIDs, and promoting health equity, integration, independence, and equality [12,13].

Several authors have examined different aspects of medical and nursing students’ attitudes toward PWIDs [6,14,15,16]. Namely, Kritsotakis et al. compared their attitudes using the community-living attitude scale–intellectual disability (CLAS-ID) short form with four dimensions: empowerment, exclusion, sheltering, and similarity. They revealed that medical students are more positive in the similarity dimension. That is, compared to nursing students, they believe that PWIDs have the same human rights as everyone else. On the contrary, nursing students showed a more positive attitude in the dimensions of empowerment and sheltering, which indicates that they support the idea that PWIDs should participate more independently in decision making but should live in sheltering facilities [6].

Furthermore, it was found that nursing students are concerned and uncertain about being prepared for communication and appropriate behavior while providing care to PWIDs [14,15]. Also, they reported a lack of knowledge to care for PWIDs and the need for additional theoretical classes and clinical practice in different settings that would allow them to experience working with this vulnerable population [14,15,16] directly. A similar attitude was held by medical students in the UK, who expressed that they were anxious and worried about treating PWIDs primarily because of the fear of communicating and interacting with them [17].

The analysis of the different attitudes and readiness to care for PWIDs, concerning the gender and age of the student, showed conflicting results [6,18,19], while in some studies, no differences were found [20]. However, in several studies, it was revealed that having a family member or a friend with an intellectual disability influenced the students’ attitudes, with those who have them being more comfortable with challenging situations [17,21,22].

Finally, Kloster et al. confirmed that nursing students are more positive toward PWIDs than the general population [23]. That finding contradicts earlier views that the attitudes of students and health professionals are the same as, or even worse than, those of the general population. Also, some findings suggest that healthcare students’ attitudes improve during their education [10]. Therefore, it is important that education addresses these biases and encourages a more inclusive and understanding attitude toward intellectual disabilities among future health professionals.

Given that the perspective from which individuals view intellectual disabilities affects how they are perceived and informs care policies and practices, this study has three aims:

1. To evaluate Serbian healthcare students’ perceptions of the rights of PWIDs, the students’ levels of social distance toward PWIDs in healthcare, and the students’ competence for providing care to PWIDs;
2. To explore whether there are differences in the students’ perceptions of the rights of PWIDs, the students’ levels of social distance toward PWIDs in healthcare, and the students’ competence for providing care to PWIDs, concerning the study program and students’ sociodemographic characteristics;
3. To assess the correlations among the students’ perceptions of the rights of PWIDs, the students’ levels of social distance toward PWIDs in healthcare, and the students’ competence for providing care to PWIDs.

## 2. Materials and Methods

### 2.1. Study Design and Setting

A cross-sectional comparative and correlational study design, using a self-assessment survey, was utilized to collect data in the Faculty of Medicine at the University of Novi Sad.

### 2.2. Sample and Data Collection

The convenience sample comprised N = 341 students (medical students n = 221; nursing students n = 120). The sample size was determined based on the total number of students from both study programs (n = 1092). Thus, using sample size software for cross-sectional studies, a sample of 280 students is required for a 95% confidence interval with a 0.05 margin of error. In order to compensate for a potential data loss of 20%, the sample needs a minimum of 336 students.

Of the 1092 questionnaires distributed, 367 were collected (33.6% return rate). Furthermore, 26 improperly filled questionnaires were considered as invalid and excluded from the study; therefore, 341 were included in the analysis (31.2% response rate).

Data were collected online using the students’ faculty e-mail addresses during the winter semester of the 2023/2024 academic year. All the students received information about the study via e-mail and a link to access the scales created in Google Forms.

### 2.3. Research Instrument

In this study, four self-designed scales based on literature data were used: (a) the general questionnaire for obtaining sociodemographic data, (b) the “beliefs about the rights of PWIDs in healthcare” scale (belief scale), (c) the “social distance toward PWIDs in healthcare” scale (distance scale), and (d) the “self-assessment of competency to provide care for PWIDs” scale (competence scale). All the scales, except for the general questionnaire, were prepared by Polish researchers, the coauthors of this manuscript [24], and they were anonymous.

The general questionnaire included the students’ gender, study program, year of study, whether they had friend or family member with an ID, and whether they had received lectures on PWID care within their compulsory and elective subjects.

The “beliefs about the rights of PWIDs in healthcare” scale (belief scale) has 14 items rated on a Likert scale, with the answers ranging from 1—strongly disagree to 5—strongly agree. Four items were reversed. The total score for this scale was calculated by summing the answers to each item. The total score ranged from 14 to 70, with a higher score indicating stronger positive beliefs about the rights of PWIDs in healthcare.

The “social distance toward PWIDs” scale (distance scale) contains ten items rated on a five-point Likert scale. The total score for this scale was calculated by summing the answers to each item and ranged from a minimum of 10 to a maximum of 50. Higher scores indicated less social distance toward PWIDs in healthcare.

The “self-assessment of competency to provide care for PWIDs” scale (competence scale) has five items evaluated on a Likert scale from 0—no competence to 4—a very high level of competence. The total score for this scale was calculated by summing the answers to each item and ranged from 0 to 20. The higher the score, the higher the competence level to provide care for PWIDs.

Pretest procedures were applied to translate and adapt the questionnaires. Adaptation involved linguistic adaptation to the cultural environment. The total score on all the scales was the sum of the responses to each item, with higher scores indicating more favorable results.

Before administering these scales in the Serbian context, a group of experts (four educators and two nursing and special education students) were asked to review the scales for clarity.

### 2.4. Data Analysis

The research results were processed and analyzed using IBM SPSS Statistics software, version 28 (IBM Corp., Armonk, NY, USA). The scale’s reliability was assessed using Cronbach’s alpha coefficient. Bartlett’s test of sphericity and the Kaiser–Meyer–Olkin (KMO) measure of sampling adequacy were applied to evaluate the data’s factorability. The construct validity of the scales was examined using exploratory factor analysis and principal component analysis (PCA) with varimax rotation.

Data analysis was performed using descriptive and inferential statistics. The normality of the data distribution was assessed using the Kolmogorov–Smirnov test. It was observed that the data distribution varied significantly (*p* < 0.05); therefore, we used non-parametric statistical analysis (the Mann–Whitney U test and Kruskal–Wallis test). Depending on the analysis, effect sizes were calculated using the rank-biserial correlation coefficient (r) or eta squared (η^2^) value.

Spearman’s rank correlation coefficient (ρ) was used to determine the degree of association between students’ beliefs about the rights of PWIDs in healthcare and their level of social distance and self-assessed competence for providing care to PWIDs. All the tests were two-sided, with a significance level of *p* < 0.05.

### 2.5. Ethical Considerations

This study was conducted following ethical principles for the protection of human subjects. Approval was obtained from the Faculty of Medicine’s Commission for the Ethics of Clinical Research at the University of Novi Sad, Serbia (01-39/48/1 of 31 May 2023). The students received a written statement explaining the purpose of the study, requesting their anonymous and voluntary participation, and guaranteeing that (non)participation in the study would not affect their further education. Informed consent to participate was obtained from all the students who participated in the study.

## 3. Results

### 3.1. General Characteristics of the Students

Most students, n = 280 (82.1%), were female, and the average age was 21.9 ± 4.1 years. The youngest student was 18, and the oldest was 48. Three-quarters of the students did not have a friend or a family member with an ID, and n = 263 (77.1%) stated that within their study program, they did not have (a) subject/s focused on PWIDs. The other characteristics of the students who participated in the study are shown in Table 1.

### 3.2. Psychometric Analyses of the Belief Scale (BS)

The Cronbach’s alpha coefficient was 0.86, with an optimal mean inter-item correlation of 0.30.

Bartlett’s test of sphericity (χ^2^ = 2713.768, df. = 91, *p* < 0.001) indicated that the correlations in the correlation matrix did not occur by chance. A KMO measure of 0.92 verified the sampling adequacy of the analysis.

The three-factor structure of the scale was obtained, and the percentage of the variance explained was 64.6%. All the individual items’ contributions to the factors were significant and exceeded 0.50, ranging between fair (0.55) and excellent (0.90) (Table 2).

Items 3, 4, 6, 7, 9, 10, 11, 13, and 14 were loaded on the first subscale, labelled “PWIDs’ general rights in healthcare”. Items 5, 8, and 12 examine the general principles of care for PWIDs, and they were loaded on the second subscale labelled, ”PWIDs’ opportunities in healthcare”. The remaining two items form the third subscale, “PWIDs’ decision making in healthcare”.

### 3.3. Psychometric Analyses of the Social Distance Scale (SD)

Cronbach’s alpha coefficient showed a high degree of reliability (0.90), while the mean inter-item correlation was 0.48.

Bartlett’s test of sphericity (χ^2^ = 2697.370, df. = 45, *p* < 0.001) indicated that the correlations in the correlation matrix did not occur by chance. A KMO measure of 0.85 verified the sampling adequacy of the analysis.

The two-factor structure of the scale was obtained, and the percentage of the variance explained was 71.5%. The contributions of the individual items were greater than 0.50 (ranging between good (0.69) and excellent (0.87)) (Table 3).

The first subscale is labelled “Providing treatment and care procedures to persons with disabilities” and includes items from 1 to 5, while the second subscale includes items from 6 to 10 and is labelled “PWIDs as my patients”.

### 3.4. Psychometric Analyses of the Competence Scale (CS)

Cronbach’s alpha coefficient showed a high degree of reliability (0.93), while the mean inter-item correlation was 0.70.

Bartlett’s test of sphericity (χ^2^ = 1530.656, df. = 10, *p* < 0.001) indicated that the correlations in the correlation matrix did not occur by chance. A KMO measure of 0.87 verified the sampling adequacy of the analysis.

The one-factor structure of the scale was obtained, and the percentage of the variance explained was 79.7%. All five items’ contributions to the factors were significant and exceeded 0.50, ranging between very good (0.81) and excellent (0.93) (Table 4).

### 3.5. Scores on the Belief Scale, the Social Distance Scale, and the Competence Scale

The scores on the scale of beliefs about the rights of PWIDs, for all the students, ranged from 26 to 69 out of 70, with a median (interquartile range) of 58 (8); the scores on the scale of social distance toward PWIDs ranged from 10 to 50 out of 50, with a median (interquartile range) of 39 (8); and the self-assessed competence scale scores ranged from 0 to 20 out of 20, with a median (interquartile range) of 10 (7).

Significant differences, concerning the study program, were observed in the students’ beliefs about the general principles of care for PWIDs, whereby medical students showed more progressive beliefs than nursing students (U = 11,356.50, *p* < 0.05, r = 0.12), and in the self-assessed competence to provide care for PWIDs (U = 10,240.00, *p* < 0.001, r = 0.19), whereby nursing students reported higher levels of competence than medical students.

In the level of social distance toward PWIDs within healthcare, a significant difference was found only by gender. Namely, female students had a lower level of social distance than male students toward PWIDs (U = 6641.50, *p* < 0.01, r = 0.15), especially in the domain of accepting PWIDs as patients (U = 6012.00, *p* < 0.001, r = 0.20). No significant differences were observed in any other variables (Table 5).

### 3.6. Bivariate Correlations Among BS, SD, and CS

Statistically significant positive correlations (*ρ* = 0.435; *p* < 0.01) were obtained between the students’ beliefs about the rights of PWIDs in healthcare (BS) and the students’ levels of social distance (SD), as well as between the students’ levels of social distance and self-assessed competence for providing care to PWIDs (CS) (*ρ* = 0.234; *p* < 0.01). The bivariate correlations between these variables are shown in Table 6.

## 4. Discussion

As the awareness of the rights of PWIDs grows worldwide, it is necessary to focus more attention on activities that would facilitate this population’s access to the services to which they are entitled, primarily healthcare services. In order to achieve this, it is necessary to understand the factors that undermine access to healthcare, including the influences of the perceptions and readiness of future frontline healthcare professionals to provide the care offered to them [25]. Therefore, this study aimed to evaluate Serbian nursing and medical students’ perceptions of the rights of PWIDs, the students’ levels of social distance toward PWIDs in healthcare, and the students’ competence in providing care to PWIDs, as well as to assess the correlations among these variables. In addition, the aim was to explore whether there are differences in the perceptions of and readiness to provide care to PWIDs, concerning the study program and students’ sociodemographic characteristics. Until now, the instruments used to assess attitudes toward PWIDs have usually been designed for the general population and, thus, are not specific for assessing attitudes toward intellectual disabilities. Therefore, this study used research instruments specifically designed for application in healthcare settings involving PWIDs. All three used scales, the belief scale, the social distance scale, and the competence scale, showed high degrees of reliability, as assessed by Cronbach’s alpha coefficients of 0.86, 0.90, and 0.90, respectively, and factor analysis confirmed their validity with significant percentages of the variance explained (64.6%, 71.5%, and 79.7%, respectively). Similar results were obtained in a study using these instruments on a sample of medical students from Poland, Serbia, and the Czech Republic [24].

The total score on the belief scale showed that future Serbian healthcare professionals recognize the rights of PWIDs to complete and easy access to different healthcare services, information, and education, which aligns with findings from previous studies [14,17,20,23,26], namely, 77.1% of the medical and nursing students strongly agree that healthcare professionals should be trained to provide care for PWIDs and 64.8% that PWIDs should have access to health information in a form that is adapted to them. In comparison, 57.5% strongly agree that PWIDs should have the right to be informed about their health. Such attitudes of students are promising because depending on cognitive functioning, PWIDs should be informed about their health, treatment, and related consequences [25]. However, only 7.8% strongly agree that PWIDs should have the right to make decisions about medical procedures, and 3.8% consider that PWIDs are generally incapable of making decisions about proposed medical procedures. These findings may be explained by the fact that more than 80% of the medical and 70% of the nursing students who participated in this study reported that they did not have a lecture focused on caring for people with disabilities.

The total score on the scale of social distance toward PWIDs indicates that healthcare students display a certain level of social distance in situations where care should be provided to this vulnerable group. This relative social distance toward PWIDs is also confirmed among underground students in the US and Turkey [27,28]. Because 85% of our students do not have a family member or a friend with an ID, they rarely have contact with PWIDs within the healthcare setting and do not gain relevant knowledge about intellectual disabilities during their course of study; this result is unsurprising [6].

The total score on the “self-assessment of competency to provide care for PWIDs” scale, which was at the mid-point level, revealed that medical and nursing students in Serbia do not consider themselves to have sufficient competence in caring for PWIDs. Namely, students reported a complete absence or a low level of competence for health assessment and the provision of treatment and care to PWIDs. The low self-assessment level of preparedness to adapt care provision for PWIDs is also noticed among Canadian medical students [29]. Conversely, almost half of the students estimated that they had a high or a very high level of competence in communicating with PWIDs, which conflicts with the findings obtained by Rozani et al. [14] and Ryan and Scior [19]. The reporting of a high level of competence among our healthcare students can be interpreted culturally and factually. Namely, in the curricula of medicine and nursing studies, the communication skills course is mandatory in the early years of study (in nursing, it is in the first year of study, and in medicine, it is in the second year).

By analyzing the differences in students’ beliefs in the rights of PWIDs in healthcare, a significant difference was observed only in the beliefs about general care principles, considering study programs, where medical students showed more progressive beliefs than nursing students. Similar results were found in a study conducted among Greek healthcare students (nursing, medical, and social work students). In that study, medical students had a more progressive attitude related to some rights of PWIDs, while nursing students predominantly had sheltering attitudes [6].

In the level of social distance toward PWIDs within healthcare, a significant difference was found only by gender. Namely, female students had a lower social distance level than male students, especially when accepting a person with an ID as a patient. This result supports the conflicting evidence that gender affects the readiness of future health professionals to work with patients with intellectual disabilities [6,18,19,20].

In addition, a significant difference was noted in the self-assessed competence in providing care for individuals with intellectual disabilities. Namely, nursing students reported a higher level of competence than medical students. This finding does not have to be surprising because nursing’s view of health is more holistic and patient oriented, while doctors are still bio-medically oriented.

Furthermore, the last results we obtained were significant positive correlations between students’ beliefs about the rights of PWIDs in healthcare and both students’ levels of social distance and self-assessed competence for providing care to PWIDs. These positive correlations confirm that the education of healthcare professionals is a key factor in overcoming attitudinal barriers. Training programs focusing on communication, empathy, and the specific needs of people with disabilities can significantly reduce negative attitudes and increase competence in providing adequate care. Continuous education and training on legislative frameworks, such as the rights of persons with disabilities, can contribute to a better understanding and application of inclusive practices at healthcare institutions [10,30].

### Limitations

Although this study explores a significant dilemma by which change can be achieved by empowering PWIDs’ rights, it has limitations in addition to its strengths. The strengths of this study are the assessment of attitudes toward only one type of disability—intellectual—as well as the participation of two student groups. The disadvantages are the single-centeredness of the study and the absence of other profiles of future healthcare professionals, such as physiotherapists and pharmacists, the study’s cross-sectional design, convenience sampling, and possible confounding factors, which limit our ability to generalize the obtained results.

## 5. Conclusions

This study is a pioneering contribution to understanding healthcare science students’ perceptions and readiness to care for people with intellectual disabilities in the healthcare sector in Serbia. Namely, our students had moderately positive beliefs about and a moderate social distance toward PWIDs and reported low competence in providing care. In addition, considering the study program, significant differences were observed in students’ beliefs in the rights of PWIDs in healthcare and students’ self-assessed competence for providing care to PWIDs, although in the level of social distance toward PWIDs within healthcare, a significant difference was found only by gender. Furthermore, significant positive correlations were found between students’ beliefs about the rights of PWIDs in healthcare and both the students’ levels of social distance and self-assessed competence for providing care to PWIDs. The first steps in achieving health equality for PWIDs are cultivating a holistic and compassionate approach to care and preventing discriminatory behavior and attitudes toward PWIDs. Therefore, it is necessary to invest further effort in redesigning the curriculum and the education of lecturers, clinicians, and practitioners.

## Figures and Tables

**Table 1:** Students’ sociodemographic characteristics (descriptive statistics).

Variable	Total (N = 341) n (%)	Medical Students (n = 221) n (%)	Nursing Students (n = 120) n (%)
Gender			
Male	61 (17.9)	50 (22.7)	11 (9.1)
Female	280 (82.1)	171 (77.4)	109 (90.8)
Study year			
First	86 (25.2)	55 (24.9)	31 (25.8)
Second	67 (19.6)	39 (17.6)	28 (23.3)
Third	85 (24.9)	44 (19.9)	41 (34.2)
Fourth	70 (20.5)	51 (23.1)	20 (16.4)
Fifth	15 (4.4)	15 (6.8)	
Sixth	18 (4.7)	17 (7.7)	
Friend or family member with an ID			
Yes	51 (15.0)	35 (15.8)	16 (13.3)
No	290 (85.0)	186 (84.2)	104 (86.7)
A subject focused on PWIDs			
Yes	78 (22.9)	42 (19.0)	36 (30.0)
No	263 (77.1)	179 (81.0)	84 (70.0)

**Table 2:** Items of the belief scale with factor loadings (three-factor structure).

Rotated-Component Matrix–PCA (Varimax Rotation; n = 341)				
	1	2	3	h^2^
PWIDs should have the right to make decisions about medical procedures.			0.739	0.672
2. PWIDs are generally not capable of making decisions about proposed medical procedures.			−0.796	0.740
3. PWIDs should have access to free preventive medical examinations.	0.832			0.694
4. PWIDs should receive all the vaccines according to the mandatory active immunization schedule.	0.788			0.621
5. PWIDs should be under sedation when undergoing medical examinations and procedures.		0.671		0.462
6. Healthcare professionals should be trained to provide care for PWIDs.	0.907			0.822
7. PWIDs should have the right to health education.	0.870			0.760
8. PWIDs receive more health services than they need.		0.662		0.467
9. PWIDs should have access to health information in a form that is adapted to them.	0.869			0.760
10. Health service utilization should be made easier for PWIDs, regardless of the cost.	0.836			0.715
11. Patients with IDs should have priority when using health services.	0.554			0.475
12. Respecting PWIDs’ dignity in healthcare is not always possible.		0.590		0.374
13. PWIDs should have the right to be informed about their health.	0.843			0.716
14. PWIDs should have privacy rights in medical care.	0.873			0.770

1 = PWIDs’ general rights in healthcare; 2 = PWIDs’ opportunities in healthcare; 3 = PWIDs’ decision making in healthcare; h^2^ = communality.

**Table 3:** Items of the social distance scale with factor loadings (two-factor structure).

Rotated-Component Matrix–PCA (Varimax Rotation; n = 341)			
	1	2	h^2^
I would accept PWIDs as patients at the hospital where I worked.	0.861		0.823
2. I would accept PWIDs as patients on the ward where I worked.	0.857		0.818
3. I would accept PWIDs as patients to whom I administer medicines.	0.796		0.653
4. I would accept PWIDs as patients from whom I take laboratory test samples.	0.874		0.773
5. I would accept PWIDs as patients whom I help with self-care activities.	0.797		0.756
6. I would accept PWIDs as patients with whom I spend my free time (breaks at work).		0.699	0.606
7. I would accept PWIDs as patients to whom I pay more attention than others to ensure their wellbeing.		0.837	0.729
8. I would accept PWIDs as patients entitled to more priority than others.		0.819	0.675
9. I would accept PWIDs as patients who engage me more than other patients in terms of activities outside the scope of my duties.		0.813	0.703
10. I would accept the need to improve my competencies, in my time off from professional hours, to meet PWIDs’ needs better.		0.686	0.611

1 = Providing treatment and care procedures to persons with disabilities; 2 = PWIDs as my patients; h^2^ = communality.

**Table 4:** Items of the competence scale with factor loadings (one-factor structure).

Rotated-Component Matrix–PCA (Varimax Rotation; n = 341)		
	1	h^2^
Health assessment of PWIDs	0.817	0.668
2. Cooperation with PWIDs	0.934	0.872
3. Provision of treatment and care to PWIDs	0.923	0.852
4. Communication with PWIDs	0.874	0.763
5. Assessment of PWIDs’ needs	0.911	0.831

h^2^ = communality.

**Table 5:** Differences, by sociodemographic characteristics, in students’ beliefs, social distance, and self-assessed competence for caring for PWIDs.

Variable	BS	BS_Subscale			SD	SD_Subscale		CS
		I	II	III		I	II	
	Mdn (IQR)	Mdn (IQR)	Mdn (IQR)	Mdn (IQR)	Mdn (IQR)	Mdn (IQR)	Mdn (IQR)	Mdn (IQR)
Gender								
Female	58.00 (6.75)	41.00 (5.00)	11.00 (3.00)	6.00 (2.00)	40.00 (8.75)	21.00 (6.00)	18.50 (5.00)	10.00 (7.00)
Male	58.00 (8.50)	41.00 (7.00)	11.00 (3.00)	6.00 (2.50)	38.00 (9.50)	20.00 (7.00)	16.00 (5.50)	10.00 (8.00)
	U = 8191.00 *p* = 0.616	U = 8411.00 *p* = 0.853	U = 8426.00 *p* = 0.869	U = 9199.00 *p* = 0.072	U = 6641.50 *p* < 0.01	U = 8077.00 *p* = 0.502	U = 6012.00 *p* < 0.001	U = 8362.50 *p* = 0.798
Study program								
Medicine	58.00 (7.00)	41.00 (5.00)	11.00 (3.00)	6.00 (2.00)	39.00 (9.00)	21.00 (6.00)	17.00 (6.00)	10.00 (8.00)
Nursing	57.00 (9.50)	41.00 (6.00)	11.00 (3.00)	6.00 (2.00)	40.00 (8.00)	21.00 (6.00)	19.00 (6.00)	10.00 (6.00)
	U = 11,676.00 *p* = 0.068	U = 12,584.50 *p* = 0.435	U = 11,356.50 *p* < 0.05	U = 13,166.00 *p* = 0.912	U = 12,335.00 *p* = 0.287	U = 12,769.00 *p* = 0.568	U = 12,292.50 *p* = 0.264	U = 10,240.00 *p* < 0.001
Study year								
First	57.00 (8.00)	40.00 (5.00)	11.00 (3.00)	6.00 (2.00)	39.50 (9.50)	20.00 (6.00)	19.00 (6.00)	10.00 (7.25)
Second	57.00 (8.00)	41.00 (8.00)	11.00 (3.00)	6.00 (2.00)	40.00 (9.00)	21.00 (5.00)	18.00 (5.00)	10.00 (9.00)
Third	58.00 (7.50)	42.00 (5.00)	11.00 (2.00)	6.00 (2.00)	38.00 (8.50)	21.00 (6.00)	17.00 (5.00)	10.00 (7.00)
Fourth	59.00 (7.00)	41.00 (5.00)	11.00 (3.00)	7.00 (3.00)	40.00 (8.00)	22.00 (6.00)	18.00 (7.00)	10.00 (6.00)
Fifth	59.00 (6.00)	42.00 (5.00)	12.00 (3.00)	6.00 (2.00)	38.00 (8.00)	21.00 (7.00)	16.00 (7.00)	10.00 (9.00)
Sixth	58.00 (6.50)	42.00 (6.50)	11.00 (2.50)	6.00 (2.00)	38.00 (6.00)	19.00 (4.50)	18.00 (8.00)	10.00 (6.00)
	H = 8.331 _df. = 5_ *p* = 0.139	H = 7.653 _df. = 5_ *p* = 0.176	H = 6.569 _df. = 5_ *p* = 0.255	H = 11.051 _df. = 5_ *p* = 0.176	H = 6.545 _df. = 5_ *p* = 0.050	H = 7.661 _df. = 5_ *p* = 0.176	H = 4.337 _df. = 5_ *p* = 0.502	H = 4.727 _df. = 5_ *p* = 0.450
Friend or family member with an ID								
Yes	57.00 (7.00)	41.00 (6.00)	11.00 (3.00)	6.00 (2.00)	38.00 (9.00)	21.00 (6.00)	18.00 (5.00)	10.00 (8.00)
No	58.00 (7.00)	41.00 (5.00)	11.00 (3.00)	6.00 (2.00)	39.00 (8.00)	21.00 (6.25)	18.00 (6.00)	10.00 (7.25)
	U = 6890.00 *p* = 0.436	U = 7239.00 *p* = 0.809	U = 7062.50 *p* = 0.605	U = 6442.50 *p* = 0.135	U = 7238.00 *p* = 0.863	U = 7015.50 *p* = 0.554	U = 7031.50 *p* = 0.574	U = 7052.50 *p* = 0.596
A subject focused on PWIDs								
Yes	58.00 (7.00)	42.00 (5.00)	11.00 (3.00)	6.00 (2.00)	39.00 (9.25)	21.00 (6.00)	18.00 (4.25)	10.50 (7.00)
No	58.00 (8.00)	41.00 (5.00)	11.00 (3.00)	6.00 (2.50)	39.00 (8.00)	20.00 (6.00)	18.00 (6.00)	10.00 (7.00)
	U = 9866.00 *p* = 0.608	U = 8905.00 *p* = 0.076	U = 9273.00 *p* = 0.193	U = 9858.00 *p* = 0.595	U = 9865.50 *p* = 0.608	U = 9779.50 *p* = 0.528	U = 9908.00 *p* = 0.647	U = 8921.00 *p* = 0.079

BS Subscale: I = PWIDs’ general rights in healthcare; II = PWIDs’ opportunities in healthcare; III = PWIDs’ decision making in healthcare. SD Subscale: I = Providing treatment and care procedures to persons with disabilities; II = PWIDs as my patients; Mdn = Median; IQR = Interquartile Range; U = Mann–Whitney test; p-values; H = Kruskal–Wallis test; df. = degrees of freedom.

**Table 6:** Bivariate correlations (Spearman ρ values) among the total BS, SD, and CS.

	BS-Total	SD-Total	CS-Total
1. BS-Total	1		
2. SD-Total	0.435 **	1	0.040
3. CS-Total	0.094	0.234 **	1

** = *p* < 0.01.

## Data Availability

The data supporting this study’s findings are available on request from the corresponding author.

## Abbreviations

The following abbreviations are used in this manuscript:

BS	scale of beliefs
CLAS-ID	community-living attitude scale–intellectual disability
CS	competency scale
KMO	Kaiser–Meyer–Olkin
PCA	principal component analysis
PWIDs	people with intellectual disabilities
SD	scale of social distance
